# Intra-operative nasal compression after lateral osteotomy to minimize post-operative Peri-orbital ecchymosis and edema

**DOI:** 10.1186/s40463-019-0370-7

**Published:** 2019-10-16

**Authors:** L. Sowerby, L. M. Kim, W. Chow, C. Moore

**Affiliations:** 1 Department of Otolaryngology-Head and Neck Surgery, Division of Facial Plastic and Reconstructive Surgery, Schulich School of Medicine and Dentistry, Western University, London, Ontario Canada; 2 Department of Surgery, Division of Otolaryngology-Head and Neck Surgery, University of Ottawa, Ottawa, Ontario Canada; 3 Department of Otolaryngology-Head and Neck Surgery, St. Joseph’s Health Care London, 268 Grosvenor St, London, ON N6A 4V2 Canada

**Keywords:** Rhinoplasty, Lateral osteotomy, Post-operative peri-orbital ecchymosis, Post-operative edema, Compression, Bruising

## Abstract

**Introduction:**

Post-operative periorbital ecchymosis and edema following rhinoplasty is a well-known sequela of surgery. Unfortunately, this can be a source of distress for patients, resulting in a longer post-operative recovery time and a delayed return to work. Trauma caused by lateral osteotomies is likely the most significant cause of periorbital edema and ecchymosis in rhinoplasty. There have been various strategies proposed to minimize swelling and ecchymosis with varying success rates and accompanied risks. Intraoperative nasal compression is one potential strategy that may reduce post-operative edema and ecchymosis with minimal risk.

**Objective:**

To determine whether applying direct lateral nasal pressure intraoperatively immediately after performing lateral osteotomies reduces visible post-operative edema and ecchymosis.

**Methods:**

A prospective, randomized blinded study on consecutive patients undergoing rhinoplasty with lateral osteotomies was conducted in a single academic tertiary care medical center. Each of the participants were randomized into direct pressure application post-lateral osteotomies on the right or the left hand side. Intra-operatively, direct lateral nasal pressure was performed on the pre-determined side for 5 min timed by stopwatch after osteotomy. Post-operatively, standard photographs were taken of the patient on post-operative days 1, 3, and 7. These photographs were then shown to 20 blinded-physicians and the degree of ecchymosis and edema was graded using a previously published scale.

**Results:**

A total of 16 patients were included in this study. Based on our blinded-grading, 11 of the 16 patients had a clear global improvement in the degree of peri-orbital post-operative edema and ecchymosis with compression post lateral osteotomies. Based on the 3 blinded expert reviewers, Periorbital ecchymosis was significantly decreased on the ipsilateral side of pressure application in 10 of the 16 patients, and periorbital edema was significantly decreased in 13 of the 16 patients. The differential degree in periorbital ecchymosis was most pronounced on post-operative day 7. Patient factors such as gender, age, skin color, history of nasal trauma, side of pre-operative nasal deviation, and smoking status did not have a significant influence on the effect of pressure application post lateral osteotomies.

**Conclusions:**

Application of direct continual lateral nasal pressure intraoperatively after performing lateral osteotomies can help reduce post-operative edema and ecchymosis up to post-operative day 7. This may lead to an overall improved appearance and subsequently an improved post-operative experience for the patient. Although the effect may be variable to some degree, this is an intervention with no additional risks involved and thus can be used in a safe manner.

## Introduction

Rhinoplasty is a widely performed surgical procedure. Unfortunately, as with all surgical procedures, there are well known risks associated with the procedure itself such as intraoperative bleeding, pain, periorbital ecchymosis and edema [1]. Such side effects, especially periorbital ecchymosis and edema may lead to increased morbidity for the patient, resulting in a longer post-operative recovery time and delayed return to work.

Lateral osteotomy, a technique used for reshaping the bony nasal pyramid, is a major contributing factor to the degree of ecchymosis and edema. This may not only have psychosocial implications and lead to a delay in resumption to social activities for the individual patient, but may also result in a loss of productivity from a societal level. In addition, depending on the degree of edema, one may also experience difficulties with vision in the early post-operative period [2].

A myriad of interventions have been studied in an effort to reduce periorbital ecchymosis and edema. These include peri-operative steroid use [2–4], lidocaine with epinephrine injections [5], fibrin sealant [6], permissive intra-operative hypotension [2, 7], and subperiosteal osteotomy techniques [8, 9]. Post-operative steroid usage has been the most extensively studied. A recent meta-analysis by Hatef et al., states that pre-operative steroids are effective in decreasing post-operative edema and ecchymosis [4]. Steroid usage, however, comes with its own risks such as psychosis, irritability, immunosuppression, weight gain, uncontrolled blood glucose and avascular necrosis of the hip. An ideal method would not only be effective in decreasing such side effects, but would also be simple, broadly applicable, cost effective, and carry with it little to no side effects. Taskin et al. recently examined the combined efficacy of intraoperative cold saline-soaked gauze compression and corticosteroids on decreasing the morbidity associated with rhinoplasty procedures [1]. This study showed that the study group, receiving compression with cold saline-soaked gauze and corticosteroids had significantly decreased periorbital ecchymosis and edema. Unfortunately, the study does not examine the role of compression in isolation, was performed in the presence of intravenous steroids and used an inter-patient control group. As such, the current study aims to examine the role for intraoperative direct compression post lateral osteotomy in reducing post-operative periorbital ecchymosis and edema using the patient as their own control.

## Materials & methods

This study was performed as a prospective randomized blinded trial including 16 adult patients (age 18 years and older) undergoing rhinoplasty with lateral osteotomies at a single academic tertiary care medical center. Approval was received from the Research Ethics Board at Western University prior to any study activity (REB# 105768). Inclusion criteria were English-speaking patients between the ages of 18–80, necessity of bilateral lateral osteotomies, and ability to attend requisite follow-up for post-operative photography as part of the study (post-operative days 1, 3 and 7). Patients were excluded if there was a history of bleeding disorders, anti-coagulant medications, residence more than 100 km from the study center (to prevent undue travel burden on patients for follow-up study visits) or if intermediate or unilateral osteotomies were required. The participants were randomized into having direct lateral nasal pressure applied post lateral osteotomies on the right or the left side using a random number generator. The allocation was concealed in an envelope and was opened at the time of osteotomy. The surgeries were performed by two consultant surgeons at the same tertiary care medical center. Intra-operatively, direct lateral nasal pressure was applied along the lateral nasal side wall of the pre-determined side for 5 min immediately after the lateral osteotomies were performed. Permissive hypotension and intraoperative steroids were used in every case and no additional changes or variables were introduced to the surgery itself or post-operative care. Also all patients who had a history of smoking stopped smoking for at least 1 month prior to surgery, and remained abstinent in the immediate perioperative period. Post-operative care for patients undergoing rhinoplasty consisted of same day discharge with a short course of oral antibiotics, analgesics, nasal saline sprays and antibiotic ointment. All patients had nasal casts and intra-nasal splints in-situ which were removed on post-operative day 7. Nasal packing was not used in any case. None of the patients were given peri-operative intravenous or oral steroids. Demographic data including age, gender, smoking status, direction of pre-operative nasal dorsal deviation, history of nasal trauma was collected as potential confounding variables. Patients remained blinded to the study group allocation throughout the study. Follow up visits were arranged for post-operative days 1, 3, 7 and at 4 weeks.

**Fig. 1:**
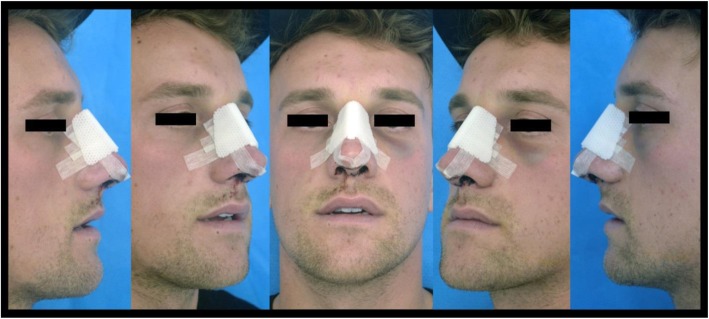
Example of a standard photograph which was taken on post-operative days 1, 3, 5, and 7. The series of photographs were shown to blinded reviewers for assessment of post-operative periorbital ecchymosis and edema. Example above was taken on post-operative day 1 and the expert ratings for ecchymosis had been 1 and 4 for the right and left hand side respectively

Standard photographs (Fig. 1) were taken of the patient on post-operative days 1,3, and 7. These photographs were then shown to 20 blinded-individuals who were not involved in the care of the patient and a global comparison encompassing periorbital ecchymosis and edema was made between the right- and left-hand sides. Each of the participants served as their own control as only the one pre-determined side had received intraoperative compression after the lateral osteotomies were completed. For further analysis on the degree of ecchymosis and edema, the standard photographs were then shown to 3 blinded-expert reviewers. The degree of ecchymosis and edema was graded for both the right- and left-hand side for each patient on post-operative days 1, 3, and 7. Previously published grading scales for periorbital ecchymosis and edema (Table 1) were utilized [1]. This utility of the two groups of reviewers were to not only capture a numerical sense on the effect of pressure application post rhinoplasty but also how this would affect one’s overall appearance in the eyes of a non-medical professional.

**Table 1:** Grading system for Ecchymosis and Edema. Adapted from previously published and utilized scale [1]

Rating	Extent of Periorbital Ecchymosis	Eyelid Edema
0	No ecchymosis	No Edema
1	Ecchymosis up to medial one-third of the upper and/or lower eyelid	No coverage of iris with eyelids
2	Ecchymosis up to medial half of the upper and/or lower eyelid	Slight coverage of iris with eyelids
3	Ecchymosis up to the full length of the upper and/or lower eyelid	Full coverage of iris with swollen eyelids
4	Entire part of the upper and lower eyelid and/or conjunctiva has ecchymosis	Full closure of eyes
5	Extension of ecchymosis beyond the malar bone	

Patient variables that could theoretically influence the degree of ecchymosis and edema were also collected. This included gender, age, skin color (Fitzpatrick grade), smoking status, history of nasal fracture, and side of nasal dorsal deviation pre-operatively. These variables were analyzed to determine whether they played a role in the recovery course for the patient, specifically in influencing the degree of periorbital ecchymosis and edema. Additionally, if the hypothesis of the effect of direct compression post lateral osteotomies were correct, we wanted to determine whether certain patient factors may influence the degree of this effect.

Data was entered and analyzed using Microsoft Excel. An unpaired T-test was used to compare the degree of peri-orbital ecchymosis between the compressed and uncompressed sides, and the Chi-square test was used to assess the differential effect of compression on periorbital ecchymosis based on the certain aforementioned patient factors. In order to evaluate the ratings from the 3 expert-reviewers, a fleiss kappa test was used to determine the inter-rater reliability. For all statistical purposes a *p*-value of less than 0.05 was considered significant. Based on the study by Taskin et al., a difference of 1 point with an average standard deviation of 1 was found be statistically significant [1]. As such, using an alpha of 0.05 and a desired power of 0.80, a sample size calculation determined a study population of 32 samples (16 patients) to be adequate.

## Results

A total of 16 patients were included in this study. Patient demographics are shown in Table 2. The average age for the patients was 35, with a range between 22 and 65. All patients underwent an open approach septorhinoplasty with 8 being randomized into having pressure applied to the right hand-side post lateral osteotomies, and 8 into having the left-hand side compressed. All of the patients were seen back on post-operative days 1, 3 and 7 for the standard photographs to be taken. No complications were reported intraoperatively or during follow up care.

**Table 2:** Patient Demographics. Patient characteristics which may influence the degree of periorbital ecchymosis and edema were recorded for a comparative analysis

Gender (M/F, n)	13/3
Age	
Range	22–65
Average	35
Skin Color	
Fitzpatrick 1–3	13
Fitzpatrick 4–6	3
History of Nasal Trauma (Y/N)	14/2
Dorsal Deviation (R/L)	9/7
History of smoking (Y/N)	5/11

The first portion of the study involved 20 blinded reviews conducting a binary comparison between the right- and left-hand side from a global perspective taking into account the overall periorbital ecchymosis and edema. The reviewers were given explanations on the study and were asked to compare the right and left sides, keeping in mind the degree of ecchymosis and edema, to have a rating for which side was worse, equal or better. There was excellent interrater reliability with a fleiss kappa score of 0.97 between the 20 reviewers. This showed that there was improvement in peri-orbital ecchymosis and edema in 11 out of the 16 patients with compression. Amongst the 5 patients that did not have an overall improvement post application of pressure, 2 patients were seen to have improved edema despite having worse peri-orbital ecchymosis and 2 patients were seen to have very similar appearance on both sides with essentially no periorbital edema or ecchymosis on either side.

The second portion of the study involved 3 blinded expert reviewers for a more detailed analysis, where the degree of periorbital ecchymosis and edema was graded for each standard photograph taken for each of the patients. Again, there was good interrater reliability seen with the fleiss kappa scores (periorbital ecchymosis K = 0.65, Periorbital edema K = 0.79). Overall, there was an improvement seen with periorbital ecchymosis with the application of pressure after lateral osteotomies in 10 out of the 16 patients, and an improvement in periorbital edema in 13 out of the 16 patients. Further analyzing the data, the difference in the scoring of ecchymosis between the two sides in each patient was calculated to determine the degree of improvement. The patients were then divided into two groups, one satisfying our hypothesis and the other satisfying the null hypothesis. On a global scheme, averaging out the 3 days there was not a statistically significant degree of improvement seen (*p* = 0.17). This was also not statistically significant on post-operative days 1 and 3 (Table 3). However, there was a significant degree of improvement seen in periorbital ecchymosis with the application of pressure on post-operative day 7 (*p* = 0.025). This is the day on which the nasal casts and stents are removed, after which patients are generally able to return to work and resume normal social activities.

**Table 3:** The difference in the degree of ecchymosis with the application of pressure post lateral osteotomies based on the gradings from 3 expert reviewers. Grading scores are based on Table 1 [1]

Differential degree of ecchymosis			
	POD # 1–7	POD # 3	POD # 7
Overall Average	1.21	1.27	1.02
Ecchymosis better on compressed side	1.48	1.57	1.71
Ecchymosis worse on compressed side	0.55	0.78	0.14
*P* value	0.17	0.12	0.025

In our subgroup analysis, looking at different patient factors, there was no significant differences found on the effect of pressure application post lateral osteotomies, based on patient factors such as gender, age, skin color, history of nasal trauma, side of pre-operative nasal deviation, and smoking status.

## Discussion

The findings of this study provide a simple, widely applicable, cost effective, and minimally invasive method of decreasing some morbidity associated with rhinoplasty.

The findings from the current study show that the application of pressure post-osteotomy may help reduce the global appearance of the periorbital ecchymosis following rhinoplasty. This may lead to an overall improved appearance and subsequently an improved post-operative experience for the patient. Using the series of standard photographs and a grading scheme for periorbital ecchymosis, the degree of improvement over the time course of recovery and its significance was analyzed. The findings show that the degree of improvement in periorbital ecchymosis with the application of pressure post osteotomies becomes significant on post-operative day 7. Understanding how a contusion evolves over time may help one comprehend why this may be. Within a contusion, hemoglobin will undergo a series of catabolic reactions as it breaks down into products such as biliverdin, bilirubin and eventually, hemosiderin. Over this time course, the external appearance of a contusion will change in its color as it gradually becomes less apparent. The grading system for periorbital ecchymosis used in the current study does not take into account the different stages of a contusion, but rather focuses on the surface area that is involved with the contusion. As such, the score does not differentiate a contusion that was at a later stage versus an earlier stage, or one that was slower to resolve. It is plausible that with the application of pressure, there is less oxyhemoglobin that is initially released into the periorbita, thereby resulting in a faster evolutionary course and resolution of the contusion. If this is indeed the case, it is possible that the current grading scheme underestimates the degree of improvement seen by the application of pressure.

In discussing the results from a more practical perspective, post-operative day 7 is when the nasal casts, stents and sutures are removed and after which patients are, in general, presumed to be able to return to work. At our institution, although the majority of patients are indeed able to return to work after this time, roughly 15–20% of the patients will prefer to stay off from work for a longer period of time. This is seen most in patients working in the customer service industry where they may be more sensitive to their appearance and its implications, such as the social stigma associated with periorbital ecchymosis. Therefore, the fact that there is a significant difference seen in the degree of periorbital ecchymosis on post-operative day 7, may be of utmost significance to the subgroup of population that would otherwise require a longer period of abstinence from social activities and work. This would translate into an improved quality of life for the individual patient, and a decreased loss of productivity for the society.

In our subgroup analysis, we were able to show that patient factors such as age, gender, skin color, history of nasal trauma, side of dorsal deviation pre-operatively, and history of smoking do not significantly influence the effect of pressure application post lateral osteotomies. Given the small sample size, there is a potential risk for a type II error. The risk of this is, however, minimized by the fact that patients acted as their own control.

Although we were able to study the effect of pressure application to osteotomy sites from an objective sense, it would be both interesting and practical to incorporate direct patient perceptions and experiences during the recovery period into the study. This may help identify certain patient factors that may make one more sensitive to the side effects of periorbital ecchymosis and edema post rhinoplasties, which may in turn help better target interventions to improve the recovery experience for specific subgroups of patients.

## Conclusion

Application of direct continual lateral nasal pressure intraoperatively after performing lateral osteotomies can help reduce post-operative edema and ecchymosis. Although the effect may be variable to some degree, this is an intervention with no additional risks associated and thus can be used in a safe manor.

## Data Availability

The datasets generated during and/or analysed during the current study are available from the corresponding author.
